# Identification of city specific important bacterial signature for the MetaSUB CAMDA challenge microbiome data

**DOI:** 10.1186/s13062-019-0243-z

**Published:** 2019-07-24

**Authors:** Alejandro R. Walker, Susmita Datta

**Affiliations:** 1 Department of Biostatistics, University of Florida, 2004 Mowry Rd, Gainesville, FL 32610 USA; 2 Currently at the Department of Oral Biology, University of Florida, 1395 Center Drive, Gainesville, FL 32610 USA

**Keywords:** Microbiome, OTU, Bacterial 16S gene, Classifier, PCA, Machine learning, Bacterial relative abundance, Random forest, Support vector machine, ANCOM

## Abstract

**Background:**

Metagenomic data of whole genome sequences (WGS) from samples across several cities around the globe may unravel city specific signatures of microbes. Illumina MiSeq sequencing data was provided from 12 cities in 7 different countries as part of the 2018 CAMDA “MetaSUB Forensic Challenge”, including also samples from three mystery sets. We used appropriate machine learning techniques on this massive dataset to effectively identify the geographical provenance of “mystery” samples. Additionally, we pursued compositional data analysis to develop accurate inferential techniques for such microbiome data. It is expected that this current data, which is of higher quality and higher sequence depth compared to the CAMDA 2017 MetaSUB challenge data, along with improved analytical techniques would yield many more interesting, robust and useful results that can be beneficial for forensic analysis.

**Results:**

A preliminary quality screening of the data revealed a much better dataset in terms of Phred quality score (hereafter Phred score), and larger paired-end MiSeq reads, and a more balanced experimental design, though still not equal number of samples across cities. PCA (Principal Component Analysis) analysis showed interesting clusters of samples and a large amount of the variability in the data was explained by the first three components (~ 70%). The classification analysis proved to be consistent across both the testing mystery sets with a similar percentage of the samples correctly predicted (up to 90%). The analysis of the relative abundance of bacterial “species” showed that some “species” are specific to some regions and can play important roles for predictions. These results were also corroborated by the variable importance given to the “species” during the internal cross validation (CV) run with Random Forest (RF).

**Conclusions:**

The unsupervised analysis (PCA and two-way heatmaps) of the log2-cpm normalized data and relative abundance differential analysis seemed to suggest that the bacterial signature of common “species” was distinctive across the cities; which was also supported by the variable importance results. The prediction of the city for mystery sets 1 and 3 showed convincing results with high classification accuracy/consistency. The focus of this work on the current MetaSUB data and the analytical tools utilized here can be of great help in forensic, metagenomics, and other sciences to predict city of provenance of metagenomic samples, as well as in other related fields. Additionally, the pairwise analysis of relative abundance showed that the approach provided consistent and comparable “species” when compared with the classification importance variables.

**Reviewers:**

This article was reviewed by Manuela Oliveira, Dimitar Vassilev, and Patrick Lee.

## Background

This present work was developed as a continuation of the work presented as part of the 2017 CAMDA MetaSUB challenge. The 2017 data was given as a pilot study of microbial communities present in samples collected from different subway stations in three American cities. In that distributed data the DNA extraction protocols and the sequencing approach were not implemented in the same or even similar manner for all three cities, and as a consequence a large percentage of samples did not yield any bacterial signal, and furthermore the experimental design was greatly unbalanced with immense disparities between the sample sizes between the three cities (1572, 134, and 18 samples). The current version of the CAMDA MetaSUB challenge data was much better with an overall small number of samples for each of the 12 cities. Although the design was still unbalanced, there weren’t large differences in the sample sizes across all cities. All datasets used in the development of this work were provided as part of the CAMDA forensic challenge by the MetaSUB International Consortium (http://metasub.org/camda-challenge-2018/). Table 1 presented a tabulated insight of the dataset for all the different groups and cities. Additionally, the DNA protocols in this challenge data had a much larger and comparable read depth, and longer pair-end reads, which resulted in better breadth, and depth of coverage of different “species” present in the DNA pool. It ultimately resulted in a raw dataset with more consistent counts across the cities, and better representation of the taxonomic hierarchy. As stated earlier, we have expanded our methodology not only to classify the mystery samples but also used better statistical inferential techniques based on the compositional data analysis of microbiome data identifying important differentiating city specific microbes. In this context, three more datasets were provided as mystery datasets through the CAMDA 2018 MetaSUB challenge to serve as testing samples for the classification problem. This work reported the results considering all the cities in the main dataset as well as the three mystery sets for the taxonomic rank “species”. As far as the open-reference picking, we included all OTUs with quality score greater than 0.5 (Please refer to Bioinformatics and Data Preparation section in the Methods section for more details). Nevertheless the large amount of zeros in the data can tell whether a species is absent in the sample or was the result of an under sampled microbiome [1, 2]. Bioinformatically, the latter issue can be improved by adding more samples [1] to the dataset or by increasing the sequencing depth [2].

**Table 1:** Number of samples included in the analyses and their corresponding city and country of provenance

Set	City	Country						
		New Zealand	U.S.A.	Nigeria	Portugal	Chile	Japan	Colombia
Training Main	Auckland (AKL)	15						
	Hamilton (HAM)	16						
	New York (NYC)		26 + 0 = 26					
	Offa (OFA)			20				
	Porto (PXO)				60			
	Sacramento (SAC)		16 + 18 = 34					
	Santiago (SCL)					20		
	Tokyo (TKO)						20	
Testing Mystery-1	Various (C1)		10 (NCY)	5	10	5		
Training Mystery-2	Ilorin (C2)			12				
	Lisbon (C3)				12			
	Boston (C4)		12					
	Bogota	No samples in the training set						
Testing Mystery-3	Various (C5)		3 (Boston)	4	4			5 (Bogota)

Table also shows the mystery sets and how the city and sets were internally coded in this work. The column corresponding to US, shows that samples from New York City and Sacramento included additional samples from the pilot analysis but those samples yielded OTUs in this present setting only in Sacramento. Light gray rows are multi-city groups where city of provenance was predicted (testing sets) for all the samples according with the corresponding training model (main or mystery-2). All samples in training sets had a counterpart in the testing sets with the exception of the city of Bogota, which has 5 samples in the testing set (mystery-3) but has no samples in the training set (mystery-2)

## Results

### Principal component analysis

**Fig. 1:**
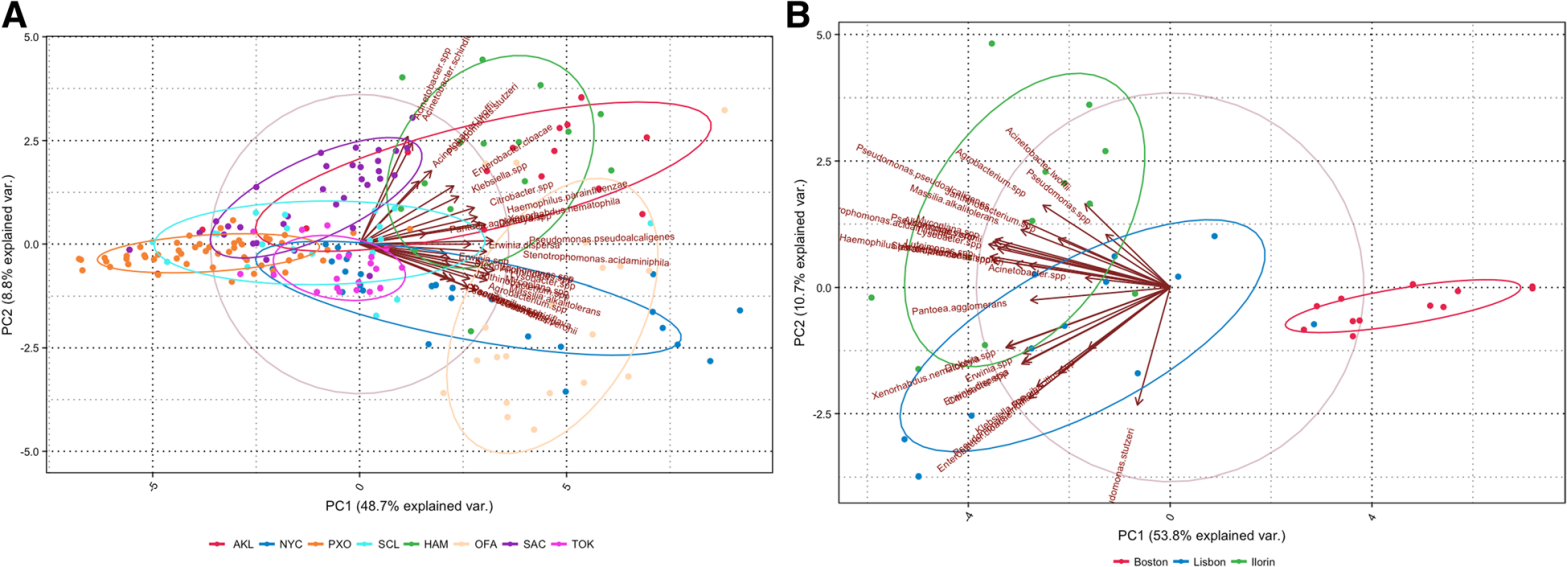
Bi-plots of first and second principal components are presented in **a** and **b** for training sets main and mystery-2 respectively. Axis labels show the percentage of the total variability in the dataset explained by the correspondent axis

**Fig. 2:**
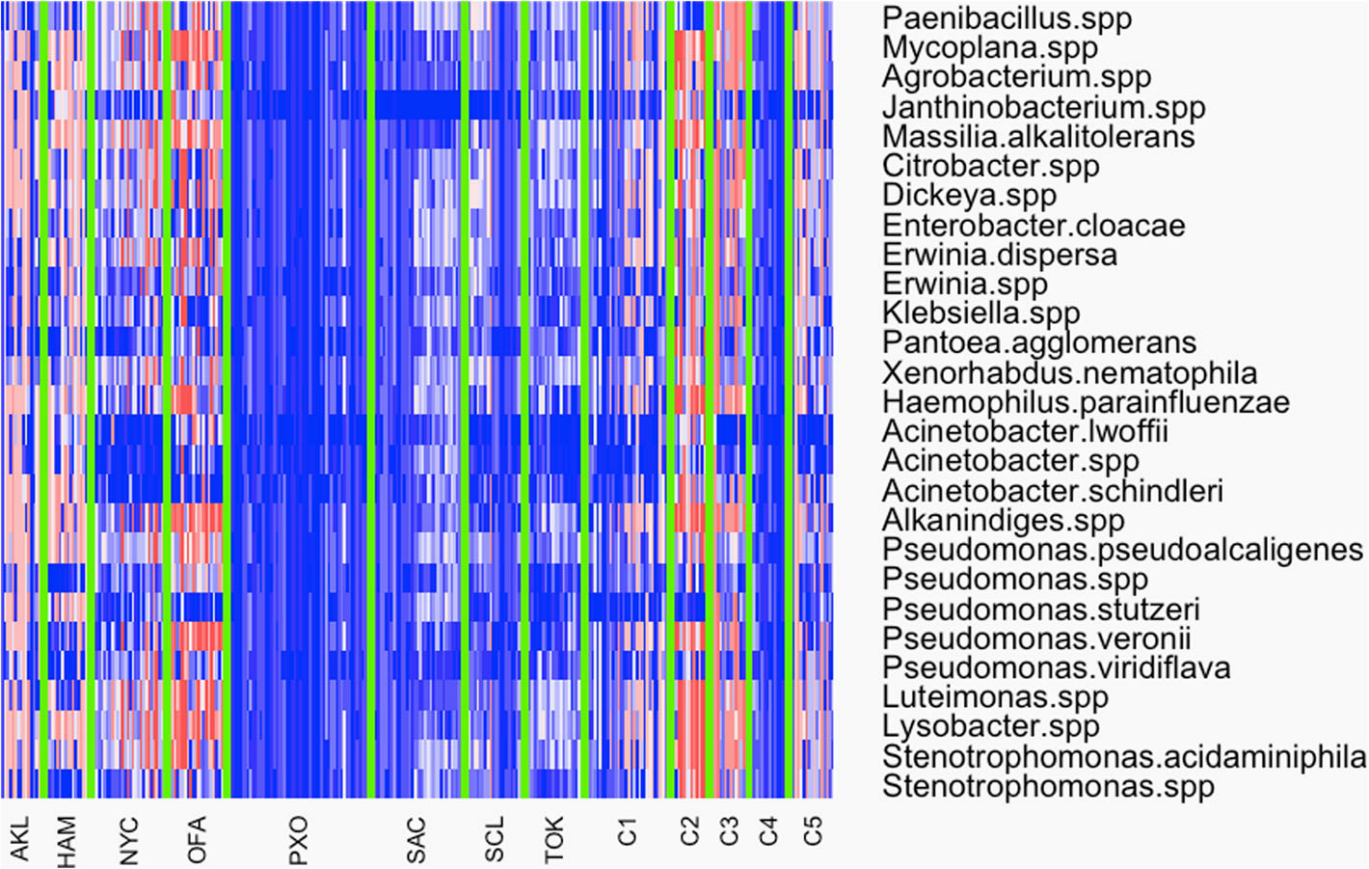
Two-way heatmap showing the log-cpm data for all cities and mystery sets for all the variables (“species”) in the main dataset. Samples from each group are separated by a light-green line to help the reader visualize the distinctive patterns shown by each set of samples. Groups C1 and C5 are testing sets with samples from multiple locations, which rendered them not showing any recognizable pattern

The PCA results in Fig. 1 shows the bi-plots for both the training datasets. Plot A depicts the main dataset and shows a better separation of the cities than the analysis in our previous work [3]. However, some city ellipses were overlapping. Specifically, Hamilton, and Auckland; both being in the same country, overlapped with Tokyo. The three cities have comparable latitude (~ 35° south and north from Equator) though the two countries are in opposite hemispheres. We observed similar overlapping between samples from Santiago and Porto, although these two are not in geographical proximity. However, overall from the plot 1A, it was evident that a large percentage of the variables (“species”) were well aligned with the horizontal axis and explained a considerable amount variability for the first principal component (48.7%). Additionally, there was a secondary set of variables, which were well more aligned with the vertical axis that also explained about 8.8% of the total variability of the data. Plot 1B presents the mystery-2 dataset (samples: C2, C3, and C4) and shows an almost perfect separation of the Boston samples, with a small overlap between Lisbon and Ilorin samples. The first two principal components explained 64.5% of total variability in the data, which is comparable with the percentage explained by the corresponding components in the main training dataset. Additionally, in B it can be seen that a group of variables was well aligned in the direction of Ilorin whereas a secondary group is aligning with Lisbon, and only a single “species” (*Pseudomonas stutzeri)* pointing down that suggests a preference towards Boston and Lisbon. A two-way heatmap of the normalized data (Fig. 2) showed that the samples from each city had a distinctive signature, which could be beneficial for the classification of the mystery samples. In the plot, the samples were separated by a light-green line to emphasize the separation of all groups, and the variables were sorted taxonomically. Obviously, groups C1 and C5 were not showing a distinctive pattern since they are multi-city testing sets. There were some cities showing similar patterns that can lead to misclassifications. However, there were others with very distinctive patterns that would be a great advantage during the classification process. In the heatmap, some “species” also showed a remarkably distinctive pattern, exhibiting a specific color in a city/s (group) and an opposite in another city/s (e.g. *Janthinobacterium spp* and *Pseudomonas veronni*), which would definitely play a key role during the classification.

### Machine learning analysis

**Fig. 3:**
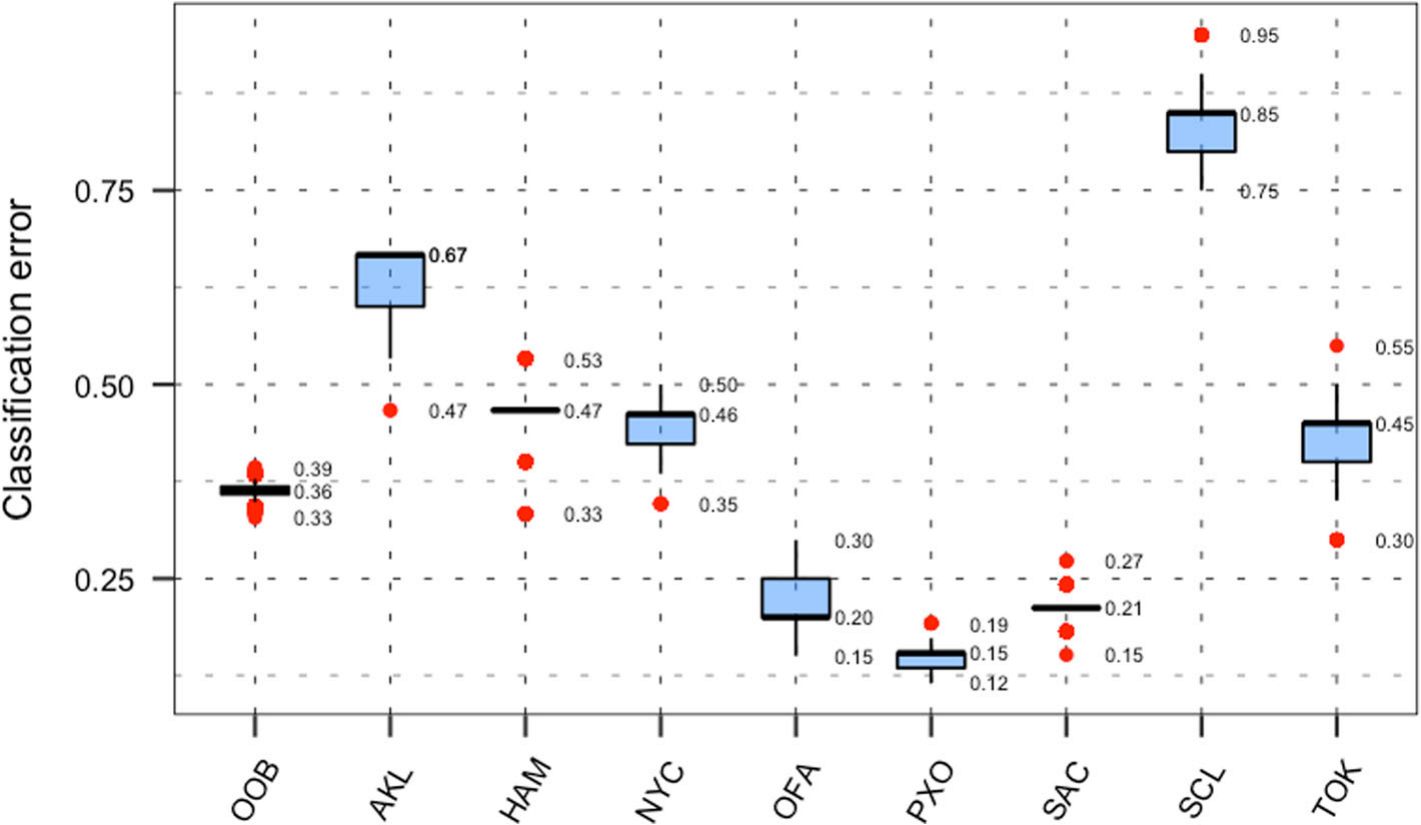
Classification error rate for the CV run with the RF classifier. Plot presents out of bag (OOB) overall classification error rate and partial error for all the cities in the main dataset (city codes can be found in Table 1)

Results from the internally cross-validated (CV) Random Forest [4] (RF) on the training set (Fig. 3) showed an over-all classification error of 36% with a narrow range (33–39%). Partial classification error rate for each city was in close range with the out of bag (OOB) error, with the exception of the city of Santiago with a median classification error rate of 85% (75–95%). The city where RF performed the best was Porto (PXO) in Portugal with a median error rate of 15% (12–19%). Better classification results for the city of Porto could be the consequence of a very distinctive bacterial signature of that city. This can be visualized in Fig. 2 where samples from this city have log2-cpm values colored with dark shades of blue which, are generally not comparable with color patterns from all other cities. Additionally, from Fig. 2 we can assess that the samples from Santiago, have overall pattern similar to samples from Sacramento and Tokyo. But the CV error rates of both cities were better compared to Santiago, which might suggest that internally the classifier encountered a distinctive “species” signal that resulted in better overall results for some cities than others. Classification errors for the mystery-2 run were better compared to the main set. The out of bag (OOB) error rate for this run was in the range of 3 and 11%, with the city of Ilorin having the best rate (~ 0%) and Lisbon as the worst ranging from 8 to 25%. Again, from Fig. 2 it can be assessed that the city of Ilorin (C2) showed a characteristic pattern for some of the “species” that was not present in Lisbon (C3) and Boston (C4). This latter also exhibited an overall pattern more in the higher range of values compared with the other two cities in the group.

Specific results for the prediction of provenance for samples of unknown origin challenge proved that the methodology implemented in this work is on the right track. Table 2 presents the final predictions of city of provenance, with RF and Support Vector Machine [5, 6] (SVM) classifiers, for all samples in mystery-1 and mystery-3 sets. It can be inferred from the table that 19 (63.3%) samples from a total of 30 samples were correctly labeled by the voted classifier in the mystery-1 testing set. Results for the mystery-3 testing set were similar with 10 (62.5%) samples, out of a total of 16, with the correct label. In this case, the testing set included 5 samples from the city of Bogota, though no samples from this city were provided in the training set. Technically, and since the classifier was not able to predict this city (samples were not included during the training of the model), the results did not show a good solution of this implementation. In this context, it could be argued that without considering the samples from Colombia, the testing set would have had 11 samples which would have raised the proportion of correctly labeled samples up to 90.9%. Comparison of the independent predictions made by both classifiers (RF and SVM) with the real labels in the mystery-1 set revealed that even though the total count of correct predictions from both algorithms are close with 19 correct labels for RF and 21 for SVM, the voted and final label only counted 19. In the mystery-3 set the total number of correct labels was 10. In this set sample C5.006 was incorrectly voted, though SVM predicted the correct label, but with a lower adjusted score than RF. All these suggested that, even though our voted method achieved a remarkably high number of correct labels, it was still not at its best. If the voting was optimized, then the final count of correct labels could go up to 23 (76.6%) in the mystery-1 set and 11 (100%) in the mystery-3 set.

**Table 2:** Final results for the classification of mystery samples from mystery set 1 and 3

Sample	Random Forest				SVM				Final Prediction	Real Label	Status of the prediction
	Prediction	Score	Departures	Adj. Score	Prediction	Score	Departures	Adj. Score			
Mystery Set 1											
C1.001	SAC	1.000	0	1.000	SAC	0.848	2	0.240	SAC	SCL	
C1.002	SCL	1.000	0	1.000	SCL	1.000	0	1.000	SCL	SCL	CORRECT
C1.003	NYC	1.000	0	1.000	NYC	0.771	1	0.297	NYC	OFA	
C1.004	PXO	1.000	0	1.000	PXO	1.000	1	0.500	PXO	PXO	CORRECT
C1.005	NYC	1.000	0	1.000	OFA	1.000	1	0.500	NYC	OFA	
C1.006	PXO	0.999	1	0.499	PXO	0.821	3	0.168	PXO	PXO	CORRECT
C1.007	SCL	0.971	1	0.471	SCL	0.769	1	0.296	SCL	SCL	CORRECT
C1.008	PXO	1.000	0	1.000	PXO	0.696	3	0.121	PXO	PXO	CORRECT
C1.009	NYC	1.000	0	1.000	OFA	0.619	1	0.192	NYC	NYC	CORRECT
C1.010	PXO	1.000	0	1.000	PXO	0.698	2	0.162	PXO	PXO	CORRECT
C1.011	SCL	1.000	0	1.000	SCL	0.741	4	0.110	SCL	SCL	CORRECT
C1.012	OFA	1.000	0	1.000	OFA	1.000	0	1.000	OFA	OFA	CORRECT
C1.013	PXO	1.000	0	1.000	PXO	0.864	2	0.249	PXO	PXO	CORRECT
C1.014	SAC	1.000	0	1.000	SCL	0.717	2	0.171	SAC	SCL	
C1.015	TOK	1.000	0	1.000	HAM	0.462	3	0.053	TOK	NYC	
C1.016	OFA	0.913	1	0.416	NYC	0.826	1	0.341	OFA	NYC	
C1.017	SCL	0.610	1	0.186	TOK	0.543	3	0.074	SCL	PXO	
C1.018	NYC	1.000	0	1.000	NYC	0.995	1	0.495	NYC	NYC	CORRECT
C1.019	AKL	1.000	0	1.000	OFA	1.000	0	1.000	Inconclusive	NYC	
C1.020	OFA	1.000	0	1.000	OFA	1.000	1	0.500	OFA	OFA	CORRECT
C1.021	AKL	0.834	3	0.174	OFA	0.997	3	0.248	OFA	NYC	
C1.022	PXO	1.000	0	1.000	PXO	0.894	1	0.399	PXO	PXO	CORRECT
C1.023	NYC	1.000	1	0.500	NYC	0.990	2	0.327	NYC	NYC	CORRECT
C1.024	NYC	0.852	1	0.363	NYC	0.898	4	0.161	NYC	NYC	CORRECT
C1.025	NYC	1.000	0	1.000	NYC	0.997	4	0.199	NYC	NYC	CORRECT
C1.026	PXO	1.000	0	1.000	PXO	1.000	0	1.000	PXO	PXO	CORRECT
C1.027	PXO	1.000	0	1.000	TOK	0.621	1	0.193	PXO	PXO	CORRECT
C1.028	OFA	1.000	0	1.000	OFA	1.000	0	1.000	OFA	OFA	CORRECT
C1.029	AKL	1.000	1	0.500	NYC	0.494	3	0.061	AKL	NYC	
C1.030	TOK	0.761	1	0.290	TOK	0.994	1	0.494	TOK	PXO	
Mystery Set 3											
C5.001	Boston	1.000	0	1.000	Boston	1.000	0	1.000	Boston	Boston	CORRECT
C5.002	Ilorin	1.000	0	1.000	Lisbon	0.754	1	0.284	Ilorin	Ilorin	CORRECT
C5.003	Lisbon	1.000	0	1.000	Lisbon	1.000	0	1.000	Lisbon	Lisbon	CORRECT
C5.004	Ilorin	1.000	0	1.000	Ilorin	0.568	1	0.161	Ilorin	Ilorin	CORRECT
C5.005	Lisbon	1.000	0	1.000	Lisbon	0.999	1	0.499	Lisbon	Lisbon	CORRECT
C5.006	Lisbon	1.000	0	1.000	Ilorin	0.616	1	0.190	Lisbon	Ilorin	
C5.007	Boston	1.000	0	1.000	Lisbon	0.749	1	0.280	Boston	Bogota	
C5.008	Lisbon	0.999	1	0.499	Lisbon	0.772	1	0.298	Lisbon	Bogota	
C5.009	Lisbon	1.000	0	1.000	Lisbon	1.000	0	1.000	Lisbon	Lisbon	CORRECT
C5.010	Ilorin	0.384	2	0.049	Boston	0.982	1	0.482	Boston	Bogota	
C5.011	Lisbon	1.000	0	1.000	Lisbon	1.000	0	1.000	Lisbon	Bogota	
C5.012	Lisbon	1.000	0	1.000	Lisbon	0.988	1	0.488	Lisbon	Lisbon	CORRECT
C5.013	Boston	1.000	0	1.000	Boston	0.998	1	0.498	Boston	Boston	CORRECT
C5.014	Ilorin	1.000	0	1.000	Ilorin	1.000	0	1.000	Ilorin	Ilorin	CORRECT
C5.015	Boston	1.000	0	1.000	Lisbon	0.750	1	0.282	Boston	Boston	CORRECT
C5.016	Boston	0.843	1	0.356	Lisbon	0.750	1	0.282	Boston	Bogota	

Table shows samples abbreviated names, partial results from both classifiers (RF and SVM) and voted results, actual label of each sample, and whether the samples prediction was correct. Results for sample C1.019 were not correct but also labeled as inconclusive since both classifiers predicted a different city with the same adjusted score. Additionally, in similar cases whether or not one of the classifiers was correct or not was irrelevant due to the inability of the pipeline to produce a label

**Fig. 4:**
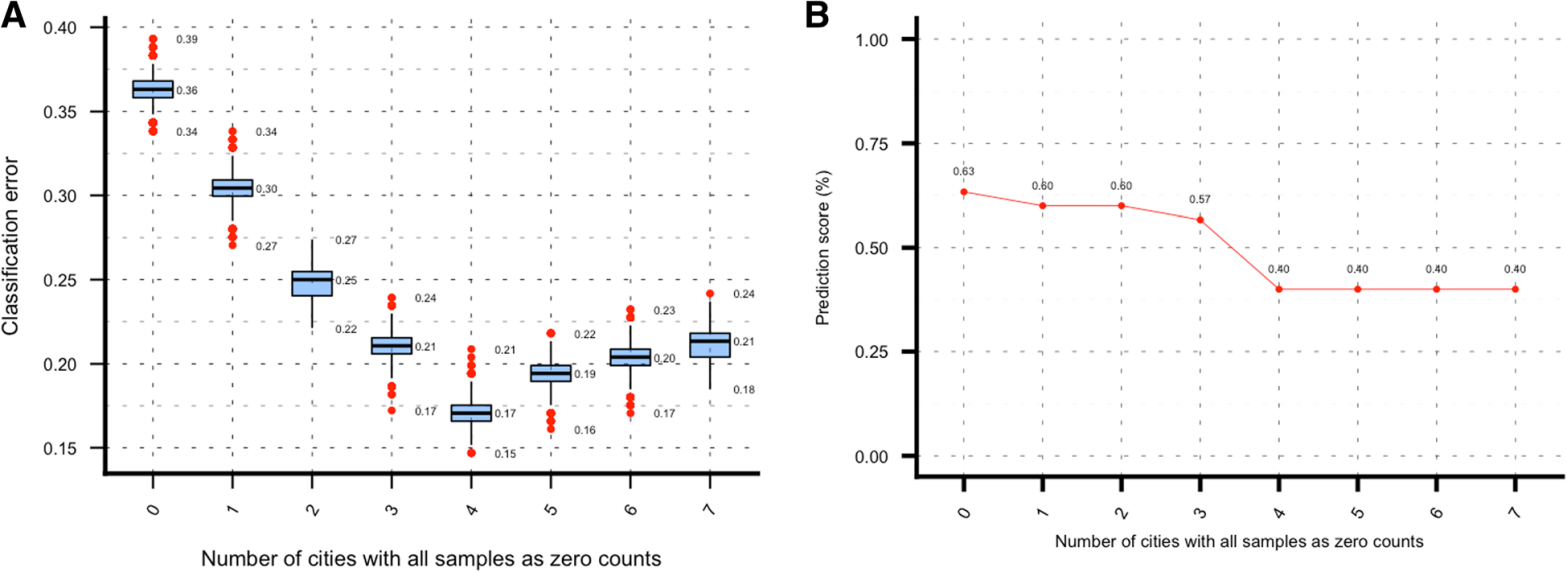
Zero-city analysis results. Plot **a** shows the OOB classification error rate for dataset with increasing number of “species” with zero-city samples (8-cities CV run after selection of the variables). Figure **b** presents the prediction score corresponding to the proportion of correctly predicted labels for the mystery-1 dataset

**Fig. 5:**
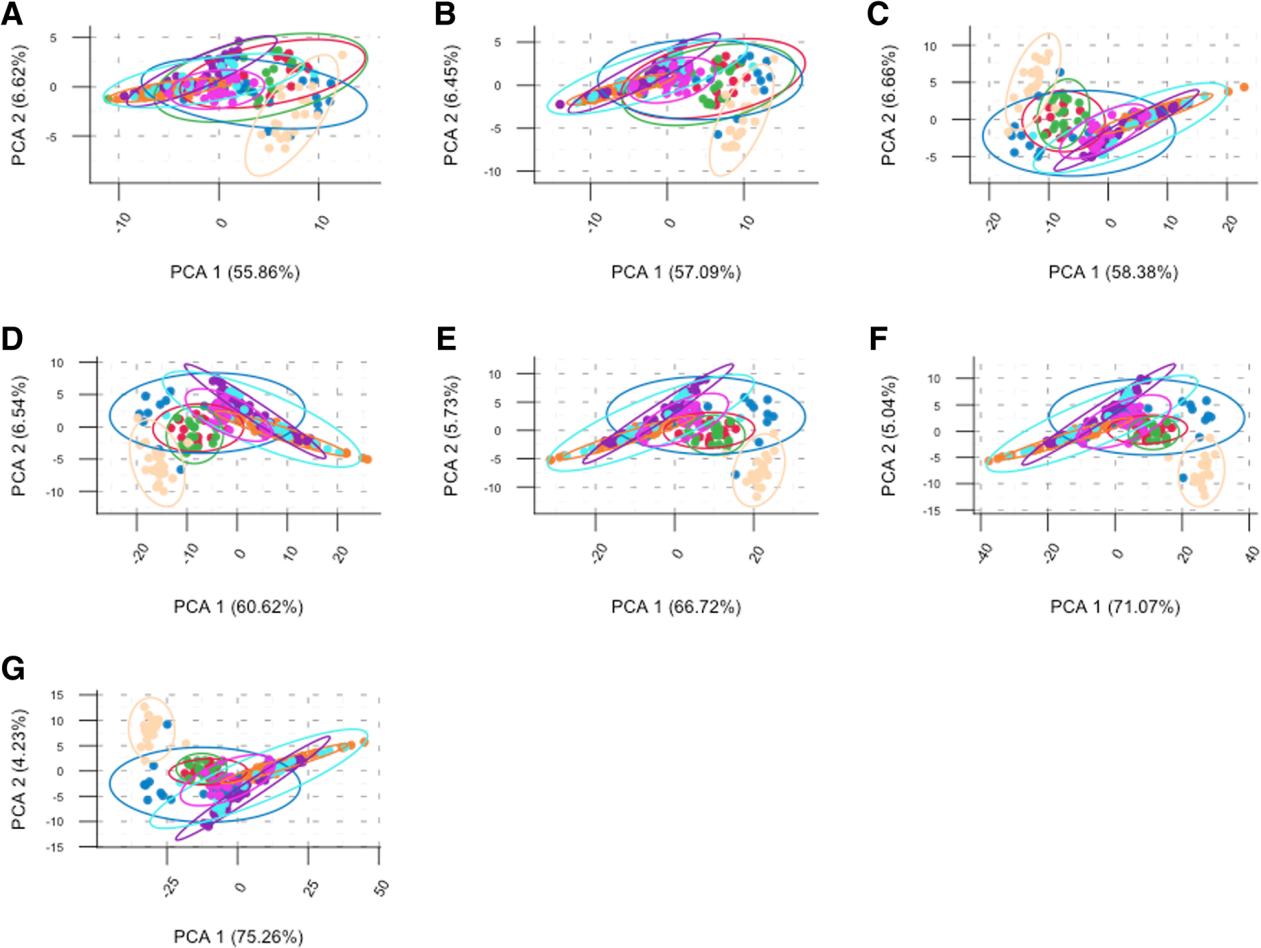
Zero-city datasets PCA plots. These plot from **a** to **g** were generated from datasets with increasing number of zero-city samples from 1 to 7 respectively. Plot also show an increasing (from **a** to **g**) proportion of the total variability of the dataset explained by the first two principal components, which can be observed also in the scale change of the x-axis. Plot A x-axis ranges from −10 to 10 whereas plot G x-axis ranges from −50 to 50

Additional results from the optimization of the datasets (zero-city analysis) were presented in Fig. 4. The left (plot 4A), depicted the OOB classification error rate for the datasets with increasing number of cities with zero-count samples in the training set, where counts “0” corresponded to the main dataset, and “7” corresponded to the dataset of 8 cities having all variables with at most 7 cities with all samples as zero counts. As evident from the plot that the error rate dropped from 36% (main dataset) to 17% when variables with at most 4 cities with zero-counts were added to the dataset. This latter statement might suggest that this was an important improvement in the generation of the dataset. However, in plot 4-B after consolidating the predictions for the mystery-1 set it was evident that the number of correctly predicted labels was continuously dropping from the maximum value obtained with the optimized main dataset. These results not only proved that the analyses presented in this work were conducted with the most optimal data possible under these conditions but also suggested that the classifier might have shown a considerable error rate reduction in the mid-range of plot 4-A. This might be due to the way the rows of zeros were added to the dataset. But the classifier failed latter when provenance labels were generated, probably because of confounding signals added by the additional zeros in the dataset and the fact that the predicted samples were not included in the training model whatsoever. Additionally, in the zero-city analysis, PCA plots were generated for each one of these datasets (Fig. 5). They progressively showed a deterioration not only in the clustering of the city samples, but also in the overall quality of the datasets, which can be visualized by focusing on how the amount of variability explained by the first two principal components were continuously increasing as the number of zero-count variables were increasing. In the PCA plot for the main dataset (Fig. 1) the total variability explained is 57.5% with the data concentrated in the range of − 5 to 5 on both axes; conversely in the zero-city analysis while adding variables, the variability changed from 62.5% in plot A to 89.9% in plot L with x-axis range changing from − 10,10 to − 100,100 from plot A to plot L.

### Differential abundance analysis

**Fig. 6:**
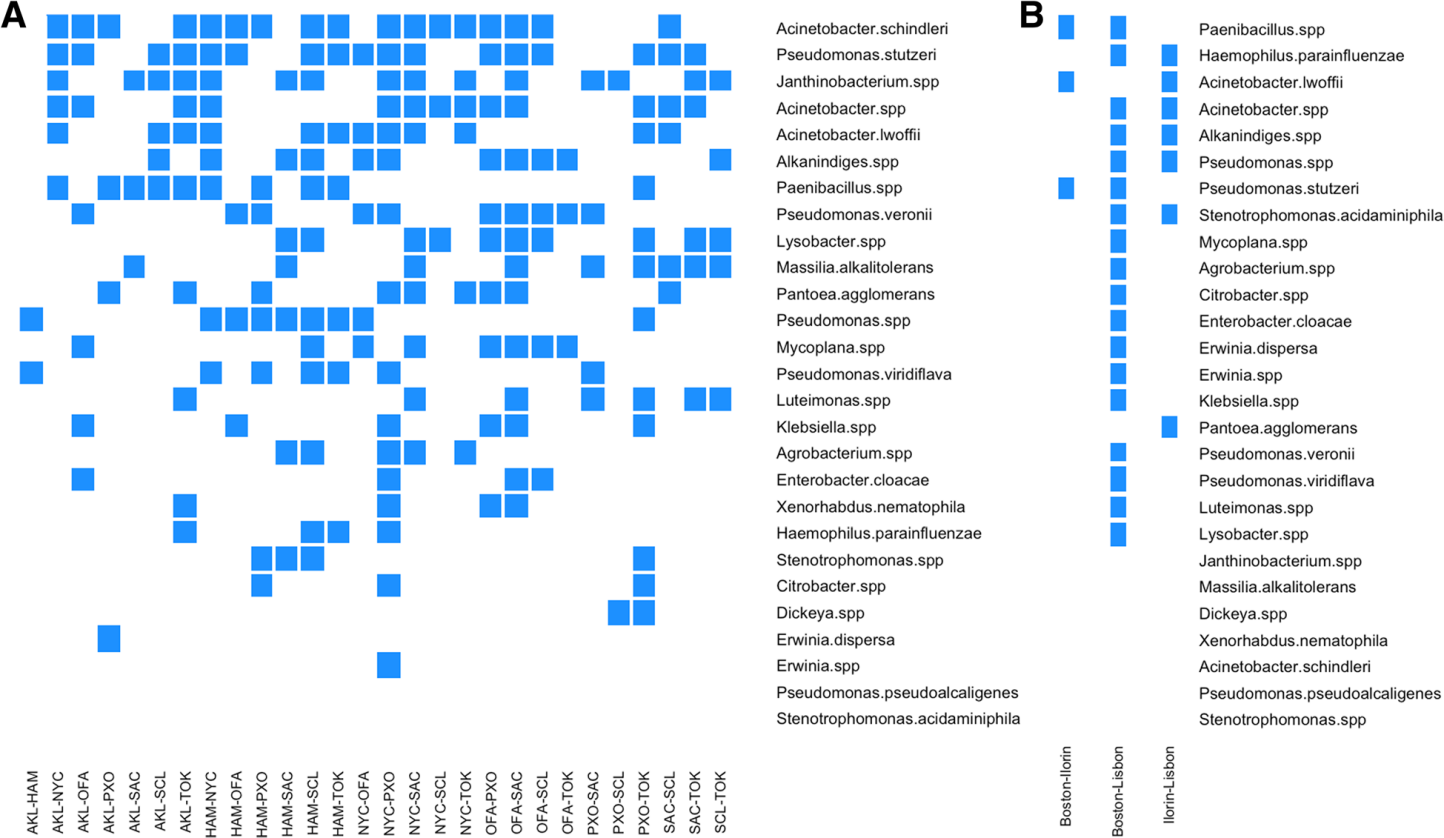
Relative abundance analysis with ANCOM results for both training sets: main dataset in plot **a** and mystery-2 dataset in plot **b**. Results are presented as significant when blue and white when there is not a significant difference in the relative abundance for any “species” in a city-by-city comparison

**Fig. 7:**
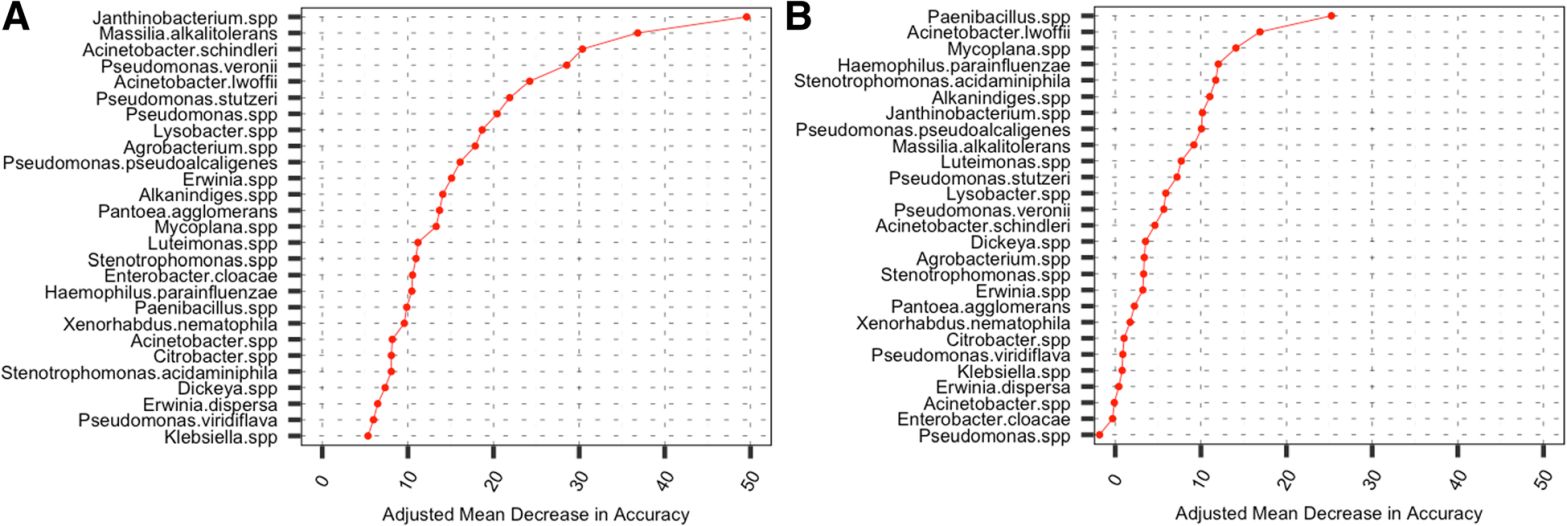
Variable importance from the CV run with the Random Forest classifier. Plot on the left (**a**) shows results for the main dataset and plot on the right (**b**) for the mystery-2 dataset. The order from top to bottom is given by the normalized score given by the classifier to each “species” at each split

Results from ANCOM [7] are summarized in Fig. 6. Plot A depicted the relative abundance analysis of “species” across all pair-wise comparisons of cities in main training set and plot B corresponds to results from mystery-2 training set. The predictors (as “species” on the right) were sorted by the number of times the normalized mean abundance was significantly different in each pairwise comparison. In plot 6-A, the top “species” in the list showed a count of 17 (number of blue squares). This means that, even though *Acinetobacter schindleri* was present in all the cities, only in 17 pairwise comparisons (total of 28 pair-wise comparisons) the abundance was significantly different. Further analysis of the ranking of the species between ANCOM results and “species” importance from RF (Fig. 7-A), showed little changes in the “species” rank between both the lists. For example, *Pseudomonas stutzeri* a bacterium belonging to the class Gammaproteocacteria, distributed widely in the environment and also identified as an opportunistic pathogen from humans [8] were present in both the lists. Another bacteria that was on top of both the lists was *Acinetobacter schindleri*, originally described by Nemec at al. [9], also belonging to the class Gammaproteocacteria. It is known to be a common bacteria present in hospitals with pathogenic potential [10]. Similarly, when comparing the “species” ranking from ANCOM results (Fig. 6-B) and classification importance (Fig. 7-B) for the mystery-2 training set, it can also be concluded that there were no dramatic changes in the relative rankings of the “species” between both the lists.

### Descriptive statistics of the dataset

**Fig. 8:**
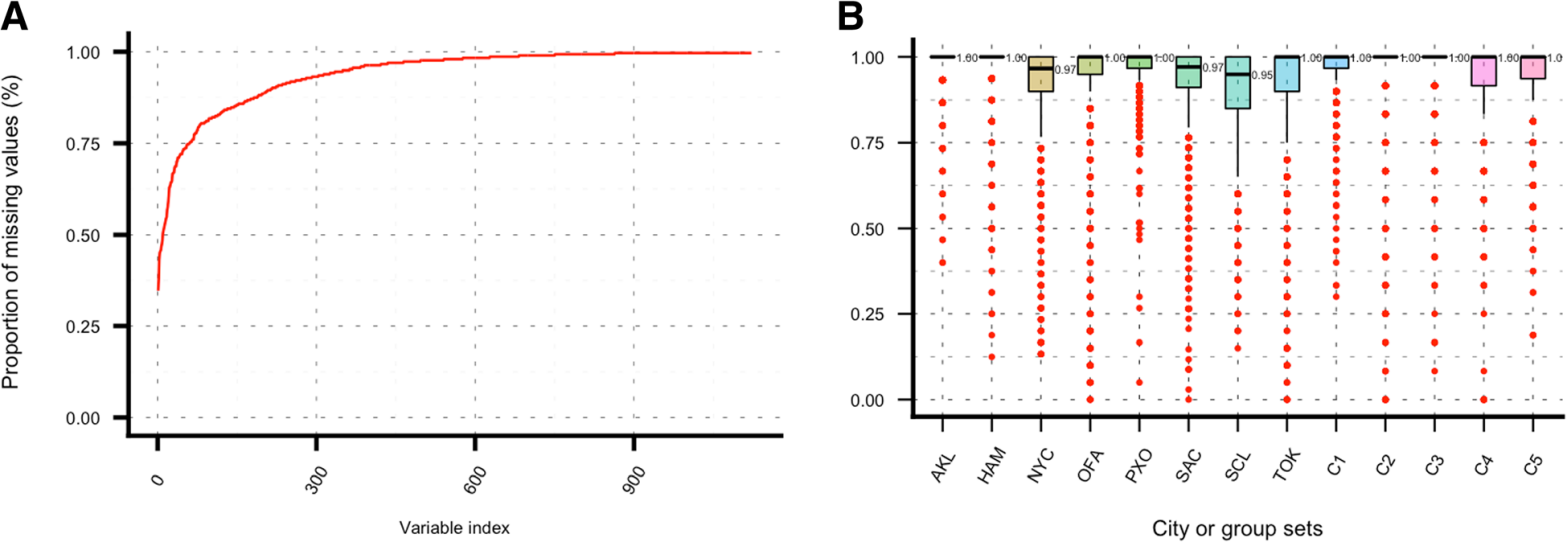
Proportion of missing data (zero count) in the dataset. Plot **a** shows the missingness found on each species (variable). The variables are sorted from less to large missingness. Plot **b** shows missingness by city (main set) or mystery samples (C1-C5). Refer to Table 1 for a better understanding of the mystery sets labels

Up to this point we have implemented the same approach to normalize and select variables we developed in our previous work. There are some changes in the machine learning implementation and how we are finding city-specific bacterial signature with ANCOM. Results from the classifiers were effectively good, but the question that remains is whether the relatively large number of zeros in the data had a negative effect in the analyses and prediction scores. Knowing the answer of this before the analysis, and even reporting the classification results without knowing the real labels of the mystery-1 set was challenging and the afterward acknowledgement of the real provenance of the samples was satisfactory considering the large percentage of samples that were correctly predicted. But at this point, one question still remains; Is it possible to improve the ~ 65% correct predictions of the samples in the mystery-1 set? The large number of zeros in the data can be graphically visualized in Fig. 8. Plot A shows the overall missingness (or zero counts) on the data presented variable by variable (species). It is highly desired that the amount of missingness do not exceed 25%. By looking at plot A we can conclude that in this work there is no such dataset with more than 25% zeros. Boxplots in plot B, are showing that for most of the cities or groups (hereafter only cities) the median (plotted as the bold black line in the middle box, and the corresponding value on the right side of each city box) is at the top of the scale. This means that 50% of the data points (in this case are bacterial counts) has only zeros. Additionally, if the city box is also shrinking towards the top, then there is a good chance that most of the species have only zeros. Only three cities departed from this trend; NYC, SAC, and SCL with a median of 97, 97, and 95% respectively. Considering this, it can be argued that the red dots (outliers in this case) are actually species with a reduced number of zeros. This also means that the actual number of variables with a decent amount of counts instead of zeros is quite limited. These results are particularly critical for both Auckland and Hamilton (New Zealand), with only 6 and 10 variables with at most 25% of zeros in the data (count of points below the 75% proportion of missing data line). These poor results for New Zealand cities might be a consequence of multiple events, such as sample acquisition, storage, under sampling, DNA extraction, and sequencing protocols [1, 2].

Additional machine learning techniques and predictions were conducted in datasets generated allowing at most 75% of missing counts in the data. Results from this analysis in the main set, were not satisfactory (data not shown in this work) with an important drop in the percentage of correctly predicted samples. We hypothesized that the low percentage of cities correctly predicted from the mystery-1 set was the result of a dramatic change in the overall presence/absence of bacterial signature pattern across all the cities in the main set. The latter reinforced results from the zero-city approach, which added variables with zeros for all samples of cities in an increasing manner, keeping the bacterial signatures of city-specific species.

**Fig. 9:**
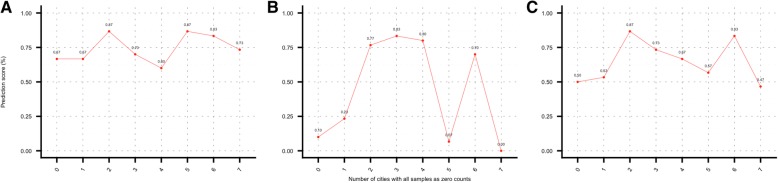
Prediction scores as a function of the number of zero-cities datasets for the main set. Plot **a** shows scores for the RF. Plot **b** shows results for SVM, and plot **c** presents combined results from the voted algorithm as described in the methods

We also tested datasets with imputed missing data (zeros). The reasoning for this was to account for missingness in the data modelling the zero-counts accordingly with the existing information from samples from the same city. For this approach we replaced all zeros in the data with NAs and run the package “mice” [11] in R for the imputations with the imputation method set as “pmm” (predictive mean matching). As we learned from previous tests, changing the bacterial patterns with this approach should have produced poor prediction scores. As a matter of fact, the percentage of correctly predicted cities was around 10%, which is extremely low and prompted us to try a different approach. Considering these results, along with what we have learnt about the zero-city datasets and the reported issues with samples from Auckland and Hamilton (see Fig. 8-B); we generated an imputed dataset only considering missingness in these two cities and generated again eight datasets by adding cities with all samples with zeros. Results from this analysis are presented in Fig. 9. As described in methods section our approach is to predict cities with RF and SVM and the vote for the “best” prediction. Plots A, B, and C in Fig. 9 present results for RF, SVM and voted predictions respectively. As it can be seen in plot C, the highest score was given by the third set (87% correct predictions), where each variable (or species) had at most two cities with all samples as zero values. These results also confirm what we already have said about the consistency achieved by RF, and its major influence in the prediction score. In Fig. 9, plot A shows that RF scores were consistently around the 75% mark for all sets, whereas SVM scores were higher for sets 3 to 5 (2 and 4 zero-cities) with a maximum of 83% for dataset 4 (3 zero-cities). It would be interesting to know if these results were affected by the way the imputation changed the bacterial signature of Auckland and Hamilton from the real patterns of these two cities or simply made these counts too divergent from the counts of the remaining cities in the main dataset, which made training more effective and predictions more accurate.

## Discussion and conclusions

This year the CAMDA challenge MetaSub dataset was of much better quality compared to CAMDA 2017, which was reflected, not only, on the amount and Phred score of the sequencing data, but also on the number of samples and cities. OTU picking in open reference mode yielded a large number of OTUs with high quality scores from the Ribosomal Database Project (RDP) classifier. Additionally, a large number of these OTUs reached the “species” taxonomy rank with a decent classification score (> 0.5). PCA analysis in both the training sets (main and mistery-2), showed that the first two components explained a large amount of the total variance (> 65%). The analysis also showed that the samples from the same city were clustered in close proximity. The machine learning analysis was effective in predicting city of provenance on unknown provenance samples and proved to be of great potential for forensic, bacterial ecology and other sciences. The city-by-city analysis of the “species” relative abundance in the main dataset (8-cities) revealed that some of them were significantly different in a large number of pair-wise comparisons. Some of these “species” were also given a high variable importance score during the RF implementation, which made this “species” highly effective during the classification. Conversely, some “species” did not show any differentiation in relative abundances across all city-by-city comparisons. Apparently, it might appear that this is a sufficient justification to remove them from the analysis; nevertheless, in specific cases these “species” were still given a relatively high importance score, which might suggest that “species” with non-significant difference in relative abundance across all cities, still can add critical signal to the data and further improve the classification results. The analysis was conducted in an optimized dataset and the results were the best achievable considering only the “species” log2-cpm as input; nevertheless, it still remains a future challenge to consider more taxonomic ranks or integration between them in the context of a machine learning approach and verify whether the class predictions might improve. Unfortunately, due to the time-restricted nature of this work we were unable to verify this statement, even though the datasets are already generated and normalized up to the taxonomic rank “order” (“order”, “family”, “genus” and “species”). Additionally, in this context it would be of great interest to implement a variable selection step with elastic-net [12], Bayes-Cpi [13], and partial least squares (PLS) [14, 15], and maybe even other approaches in order to conduct a more analytical and inclusive selection of not only “species” but considering all the taxonomical variables generated during the OTU picking with QIIME [16].

Regarding the implementation of the classifiers in the code, this can still be optimized by training the models in the same subset. This would lead to more comparable, and possibly better results. Also, the voting algorithm definitely requires more work in order to achieve a perfect voting score. Even though the voting score between RF and SVM was high in both the testing sets, there were still a small number of samples (5 out of 46 from both datasets) that were incorrectly voted but correctly predicted by one of the classifiers. As it was stated before, this was not at all a sensitive issue since the voting was quite effective, though still not perfect. More research would help to improve the voting mechanism. Regarding imputation of zero-values, it is not recommended to impute all the missingness in the incomplete dataset, because this would effectively change the bacterial patterns of city-specific species, which can, ultimately, lead to misclassification. Alternatively, as it was shown, imputing specific cities (in this case Auckland and Hamilton), resulted in an increase of 24 points in the percentage of correctly predicted cities. As for this work we only used “pmm” as imputation, though there are some other methods within the “mice” package that can still be applied. Finally, as a closing remark, the results presented in this work showed an effective method to process, optimize, and classify the metagenomic samples by origin, but still there are scopes to improve upon the results by carefully adjusting for all the possible sources of errors in such data.

## Methods

The design of this analysis was highly prompted by the experience acquired from the CAMDA 2017 MetaSUB Challenge [3]. The main factor influencing the changes of the analytical procedure and the results obtained was the quality of the sequencing data, which allowed us to apply a uniform quality filtration scheme. The open reference OTU picking with QIIME [16] was now incredibly successful and effective that yielded a large number of features reaching the taxonomic rank “species”, which was barely seen in our previous pilot analysis, since a limited number of “species” exhibited a taxonomy score greater than the stablished threshold. Considering the compositional nature of the count data, we implemented a normalization step, which took into consideration not only the structure of the data, but also the experimental design and number of samples. Finally, we run analyses using unsupervised and supervised techniques. A more detailed description of these implementations can be found in the following sections.

### Bioinformatics and data preparation

New sequencing data provided as Illumina MiSeq paired-end reads, was screened for Phred score. This analysis revealed that this data was of a much higher and consistent quality compared to the 2017 sequencing dataset provided by CAMDA 2017 MetaSub data. Additionally, in order to remove all human DNA sequences from the samples, and to reduce the size of the FASTQ files in the process, a small subset of samples from each country were mapped to the human genome reference (accession number: GCA_000001405.1, http://hgdownload.cse.ucsc.edu/goldenPath/hg19/chromosomes/) with BWA [17]. Later with Samtools [18] and Picard (http://broadinstitute.github.io/picard) we extracted the unmapped sequences, supposedly containing only microbial and bacterial DNA. Ultimately, by analyzing this filtered sequencing data we realized that the contamination by human DNA in the samples was not enough (< 1%) to justify the implementation of this step in all the samples. The results from this screening was a key factor when designing the quality filtering approach further in the bioinformatics part of the pipeline. At this point it is virtually unfeasible to better evaluate other sources of contamination in the samples, which would depend on sample storage, DNA extraction protocols, sequencing technology, biology grade water, DNA extraction kits, amongst other sources [19–21].

Phred score filtering was implemented with FASTX-Toolkit [22] with the purpose of setting a uniform quality standard for all cities, removing low quality reads, and reducing the size of the FASTQ files in order to keep the computational burden in check. The parameters used in the filtering were q = 38 as a minimum Phred score to keep and *p* = 50 to set a minimum percentage of the bases that must have a quality score of 38. As previously stated, we performed a preliminary quality screening of the sequencing data which revealed that all cities shared high quality Phred scores and have long reads. The latter was a reasonably good justification for applying this filtering scheme for all cities without changing the parameters. The filtered data was then transformed in a FASTA format and finally parsed to QIIME [16] to perform an open reference OTU picking and later a taxonomy assignment with the RDP classifier [23]. After OTU picking, all the counts with quality scores (calculated by the RDP taxonomy classifier) smaller than 0.5 were removed from further analyses. The resulting filtered OTUs were aggregated adding the corresponding counts for each existing taxonomic rank given by the classifier. Aggregated raw counts were normalized using the “R” function “voom” [24] (included in the package “limma” [25]) to generate log2-cpm, which guaranteed that counts are bounded away from zero to make the logarithm meaningful. The normalized data was maintained for all features that were given a “genus” and a “species” taxonomy assignment during the RDP run. Finally, the variables (created by concatenating the corresponding names of ranks “genus” and “species”) were selected such that at least one sample, within each city, should have had a count greater than or equal to two, which was the minimum count possible for any OTU given by QIIME (non-zero counts). This would ensure that the variances for all variables across cities were always non-zero. We also generated the datasets for more taxonomic ranks in order to determine their usefulness in achieving our goals; results which are not reported in this work. Additionally, with the purpose of validating how the final dataset was chosen, we ran the classifiers on datasets containing an increasing number of variables with all samples per city only with zero-count (we called this the “zero-city” analysis). A better elaboration on this idea was that, in our previous work we only considered the dataset with all the variables where at least one sample was non-zero in a city (as described before). However, we did not further tested what would have happened if we tried adding variables with zero-counts in all the samples or even testing the full dataset. In this work, we have generated seven additional datasets by subsequently adding more variables with the following rule: the second dataset was generated by adding to the first (or main dataset) all the variables with zero-counts in only one city, considering that it wouldn’t matter which was the zero-count city. The third dataset was generated by adding to the second set all the variables with zero-counts in two cities. The other datasets were generated following this rule until, finally the algorithm added all the variables with only zero-counts in all cities. Obviously having a large number of variables with zero-counts in the data is not ideal since it would create an increasing problem with the variance estimation, but the purpose of this exercise was to empirically proof that our approach and the construction of the dataset was optimized at its best.

### Statistical analysis

All further statistical analyses in this work were conducted in R [26] environment (version 3.3.2 “Sincere Pumpkin Patch”). First, we conducted an unsupervised PCA analysis in order to validate the quality of the dataset by checking its consistency and the samples-by-city clusters. Additionally, we generated heatmaps of the data to visually asses their potential for predicting city of provenance. The supervised method was implemented as a voted machine learning approach with two well regarded classifiers, namely Random Forest and Support Vector Machine. These two algorithms were implemented independently to predict provenance and ultimately were voted accordingly as explained in the machine learning section.

### Principal components analysis (PCA)

Unsupervised analysis of normalized data was conducted on the bases of correlation structure of common “species” found across all cities in the main, and mystery-1 datasets. Eigenvalues were used to calculate the variability accounted for each component. Two-dimensional bi-plots and three-dimensional (not presented in this manuscript) plots of the first three components were generated to assess the group separation of the cities. Additionally, we plotted two-way heatmaps of the variables (“species”) for all cities in order to visualize various bacterial signature patterns across all cities (samples). The PCA analysis was also implemented in the additional datasets containing increasing number of zero-count (zero-city datasets) samples across cities as described in the Bioinformatics and Data Preparation section.

### Machine learning analysis

The machine learning analysis was conducted at this stage running two classifiers: Random Forest (RF) [4], and Support Vector Machine (SVM) [5, 6]. RF was implemented with 1000 trees and 20 variables chosen at each split. We have fitted the model for all the samples in the main set (8 cities) and consider this the training model. From this cross-validation (CV) type run we recorded the overall out-of-bag (OOB) classification error as well as the by-city error rates considering only the samples from the eight known cities. We also recorded the variable importance computed by the classifier in the training model. After fitting this training model, we predicted the city of provenance of the samples from the mystery-1 set. We recorded the predictions and we repeated this process 10,000 times.

For mystery sets 2 and 3 we conducted a similar implementation having the mystery-2 set (3 cities with 12 samples each) for training the model and the mystery-3 set (16 samples) for predictions. Again, we repeated this cycle 10,000 times and recorded the results accordingly as we did with the 8-cities and mystery-1 sets.

The SVM classifier was implemented in a similar manner with some small variations due to the intrinsic nature of this approach. Fitting of the training set was conducted in a 5-fold-city CV scheme for both, 8-cities and mystery-2 sets. This would randomly drop a number of samples from each city to generate the training set. The cost of mis-classification was set in 1000 and the gamma parameter was set as default (gamma = 1/#variables). After fitting the model with the training set, predictions of city were done for the corresponding mystery set. This process again was repeated 10,000 times. No prediction of the excluded samples from the training sets were generated and reported.

After the predictions were all done we consolidated the results as number of times a city or cities was or were predicted for each sample in the testing set (mystery-1 and mystery-3) and we calculated a prediction score as, the number of times the city with the highest count divided by the total number of repetitions (10,000), which reflects the proportion of hits. Additionally, we recorded the number of cities that were predicted for each sample. In this work we are proposing an adjusted score to decide whether RF or SVM predicted is the final voted city. The prediction score was then multiplied by the ratio between the score itself and the number of departures. The number of departures was the count of cities that were predicted for any particular sample (this is an attempt to adjust the prediction score with the total number of cities predicted for each sample). Finally, we voted for the label predicted by the classifier with the highest adjusted prediction score. This implementation was also conducted in the zero-city datasets and the results were presented accordingly in the Results section.

### Differential abundance analysis

Bacterial abundance analysis for the normalized log2-cpm was conducted with the analysis of composition of microbiome data by the ANCOM [7] package in R. This method was proposed to account for the compositional nature of microbiome data and fitted well with the underlying structure of our own dataset. Twenty-eight pairwise comparisons were made for all combinations of the eight cities in the main dataset. ANCOM level of significance was set to 0.2 and the output was a list of the variables that were significantly different for each pair of cities. Results were summarized as the number of times the abundance of a “species” was found to be significantly different across all pairwise comparisons. This count later was compared with the “species” importance given to the variables during the classification analysis. This analysis was also conducted for the mystery-2 (3 cities set).

## Reviewers’ comments

### Reviewer’s report 1: Manuela Oliveira

Reviewer’s comments: Several aspects concerning scientific accuracy, methods description, Figures and ethics should be a addressed previously to consider the manuscript for publication. Methods: ­ more information should be provided about the samples (mainly where ­ with the indication of GPS coordinates ­ and when ­ I supposed these samples where collected in 2016) ­ more information about DNA extraction and sequencing should be provided ­ more information about the results (e.g.: alpha­ and beta­diversity) should be provided Figures: ­ Image resolution should be improved. Ethics: ­ I am sure that the MetaSub project received more founding that the one indicated in the paper ­ No reference was made to the MetaSub Consortium ­ There is an agreement with the Portuguese companies that manage these subway systems (Metro do Porto and Transportes de Lisboa) that data cannot be published with the previous consent from this companies. This approval should be presented in the “Ethics approval and consent to participate” or “Consent for publication” sections.

*First we want to thank the reviewer for the valuable comments and overall evaluation. The work presented in this manuscript is part of the CAMDA 2018 challenge, and the samples included on these analyses were given to the participants as part of the MetaSUB Forensic Challenge. The metadata provided contained information related to the provenance of the samples, and the type of surface the samples were collected from. Unfortunately there was no information regarding the sequencing technology, DNA extraction protocols, and GPS coordinates. We are sure that information exists, but for the purpose of the competition we were supposed to use only part of the data provided for the challenge for CAMDA 2018. All the images were generated in high resolution prior to the submission to the journal. Regarding the reviewer suggestion to present more results (alpha and beta diversities), we can say that we have reported those as part of the experience acquired in the 2017 CAMDA challenge. However, that information is not really necessary when considering the current objective of this work. Finally, thanks again to the reviewer to bring our attention to the fact that we have failed to mention the MetaSUB International Consortium. We sincerely apologize for this omission, which has also been corrected in the manuscript with the appropriate mention of the sample source. Finally, regarding the use of the data originally provided by the consortium, we can declare that there is no conflict or consent to publish issue regarding these results as the data was provided to the participants as a part of the CAMDA 2018 challenge in agreement with the MetaSUB International Consortium.*

### Reviewer’s report 2: Dimitar Vassilev

Reviewer’s comments: 1) There are some textual inconsistencies like wrong words (“rage” instead “range”) etc., some unnecessarily long sentences (67 lines). 2) The most frequent problems in the presented text are in the notations and abbreviations such as: Phred score or Phred quality, RDP classifier, PLS, bash scripting.

*Thanks to the reviewer’s for his valuable comments and the overall assessment of the manuscript. Also thanks for catching the “rage/range” issue, which was corrected accordingly, as well as references to Phred quality score, RDP classifier and PLS regression. We have fixed all of them.*

3) The most important problems are with explanation of the methodological approaches (PCA) and (RF, SVM) for validation why such approaches are used and what they can solve for the purposes of the particular results. As we know the PCA can help in interpretation of the data but will not always find the real patterns. In this line I think that the use of classical PCA is somehow problematic in the case of the study in particular for the analysis of such unbalanced count data which are variable and contain outliers. I would suggest the use of robust PCA (Reference: Introduction to Robust Estimation and Hypothesis Testing (Statistical Modelling and Decision Science)), also and to comment the difference between the two methods with the aim how the PCA will group in a better way the samples and how the quality of this grouping can be validated by the RF classification. Also the authors should comment the correlations (Pearson’s and robust) together with the obtained biplots. On the other point when explaining the used RF model authors must comment the advantages: decorrelates trees relative to bagged trees (important when dealing with multiple features which may be correlated) and the reduced variance (relative to regular trees) which is beneficial for the purposes of the study and disadvantages that RF is not easy to be interpreted visually. There also be such comments and explanations for the reason to use the SVM.

*The use of robust PCA in this work may result in interesting new information, unfortunately at this point we are unable to pursue that. The reasons are many, though one of the most important is that it seems to fall a little out of the scope that we wanted to achieve in this work, which was underlined by the objectives given in the CAMDA 2018 forensic challenge. There is no doubt that in future projects we definitely will consider this suggestion.*

4) About the further improvement (methodological) of the analysis my suggestion to the authors is to have in mind methods based on zeroinflated models (for such unbalanced, rich in zeroes data) and obviously spatial (geospatial) models for analysing the microbial data distributions with some criteria for testing and fitting of the models. 5) The references used in the study can be improved by referring the sources (sites) of all the methods, software, etc. in the study. My suggestion to the editorial board of Biology Direct journal is the submitted material to be accepted after considering the related remarks and comments.

*These suggestions are again very interesting and the use of zero-inflated models can be a really interesting solution in order to deal with the zero-counts. This can result in a more robust dataset that not only would include the common variables across all the cities, but all others. Such data can open new perspectives in order to really search for those unique “bugs” across the different locations. This can also result in an interesting spatial analysis, but again for this work fall significantly far from the project objectives and the timeline we were given to develop the work and manuscript. As a closing remark on the zero-inflated data, the preliminary analyses revealed that the normalization of the data, which included the experimental design, did not have a strong effect on the quality of the predictions when using the full dataset. This is the reason for excluding those variables with high counts of zeros (refer to pages 14–15 for more details).*

### Reviewer’s report 3: Patrick Lee

Reviewer’s comments: 1. The authors should take the opportunity to compare the strengths and weaknesses of the two algorithms for the purpose of identifying the mystery samples.

*First of all we thank the reviewer for the valuable suggestions and evaluation of the manuscript. Regarding this first comment, if the reviewer is referring to the random forest (RF) and support vector machine (SVM) classifiers, we understand the reasoning behind the suggestion, but we also believe that it would be beyond the scope of the competition.*

2. The challenge in 2017 suffered from the problem of not having enough samples. Although there were mores samples for the 2018 challenge, the authors should test what is the minimum number of samples required for both algorithms to perform adequately and how the identification accuracy varies as the number of sample increases.

*Well that is not really what happened. The 2017 data was highly unbalanced a large number of samples in one city and a very small sample size for others. Additional problem was the fact that there were only three cities and the sequencing approach to generate the data was also not the same in one city, hence the amount of sequencing data was also highly unbalanced. In this work we were given samples from 8 cities (plus a number of additional mystery samples). In this year challenge, the design was still not balanced but the sample sizes were more similar between the cities and the sequencing approach was comparable across cities, which resulted in a more robust dataset, analyses, and results.*

3. P. 17. Please further explain the rationale behind the adjusted score to decide whether RF or SVM should be the final answer. Has this approach been used elsewhere and what is the basis for the calculation?

*There are many publications reporting voted algorithms and in our work the voting was mostly driven by the proportion that a city was voted and the counts of cities predicted for a sample. This is a simple voting mechanism and, as it was stated in the manuscript, this voting mechanism can still be improved upon. We have refered to this on Page 12 of the manuscript. However, as far as the biological conclusions presented in this work the voting mechanism still have produced really promising results.*

4. P. 8, Figs. 6 and 7. It seems many signature species are commonly found in the environment but there are not a lot of human­associated species. Given that these are samples from the subway, one would expect more human­associated species. Please further discuss the implications of this result and the lack of unique or highly specialized species that one might expect are only found in a specific city or region (e.g., Auckland in the Southern hemisphere).

*The dataset used in this work was generated by choosing all the variables (species) that were “detected” in at least one sample in every one of the cities (please see pages 14–15 for more details on how the dataset was generated). This approach was also implemented in a similar fashion in our 2017 work. The justification for this is that the zero-inflated data was not giving good prediction results and the PCA plots were showing a linear pattern for each city (this is not a good sign). This is probably one of the reasons there are not human-associated species in the data. This suggest that their abundance is not really high enough to pass the zero-counts threshold for selection. This is the reason why we thought that the zero-data analysis was important since in a controlled way was including variables that were present in most of the cities.*

5. For the signature species identified, what are their relative abundance in the different cities? Will be good to have a figure showing this result.

*In our 2017 work on metagenomics we went in this direction, but we believe that doing so, departs from the objective of this work. We are interested in finding the bacterial signature present in different cities and use this data in a machine in order to generate results. This work does not intend to go into a population based metagenomic analysis.*

6. Have the authors actually identified city­specific species? That is, are there species unique to a particular city and not found elsewhere? If not, the authors should consider revising the title.

*Again this is not our objective. If we go to the full dataset, before selection we would be able to call for city-specific species. During early testing of this methodology full datasets were giving poor prediction results, because the dataset was heavily loaded with zero counts. The title suggest that we are looking for important bacterial signature not city-specific species abundance. The difference is obvious and for our purpose we are interested in those variables that are present in most of the cities in relative abundances that can make the difference between bad and good predictions.*

7. The quality of all the figures need to improve and the writing can use further polishing

*The image resolution was set in 300 dpi, which is more than enough for publications. We have realized that the editor manager program generates a PDF file with the images in low resolution, which sometimes is more than enough to follow the text. Additionally, the file also contains a link to download a full resolution version of the image as needed. Regarding the writing polishing, we have made some editing to further improve the manuscript; particularly correcting some abbreviations, inconsistencies, and other minor issues.*

## Data Availability

The datasets supporting the conclusions of this article can be obtained from the CAMDA 2017 website http://camda2017.bioinf.jku.at/doku.php/contest_dataset

## Abbreviations

ANCOM: Analysis of composition of microbiomes
ANOVA: Analysis of Variance
NGS: Next Generation Sequencing
OOB: Out of bag
OTU: Operational Taxonomic Unit
PCA: Principal Component Analysis
PLS: Partial least squares
RF: Random Forest Classifier
SVM: Support Vector Machine
WGS: Whole Genome Sequencing

## References

[1] Hughes JB, Hellmann JJ, Ricketts TH, Bohannan BJM. Counting the uncountable: statistical approaches to estimating microbial diversity. Appl Environ Microbiol. 2001. 10.1128/AEM.67.10.4399-4406.2001 PMC93182 11571135

[2] Sohn MB, Du R, An L. A robust approach for identifying differentially abundant features in metagenomic samples. Bioinformatics. 2015. 10.1093/bioinformatics/btv165 PMC4495302 25792553

[3] Walker AR, Grimes TL, Datta S, Datta S (2018). Unraveling bacterial fingerprints of city subways from microbiome 16S gene profiles. Biol Direct.

[4] Breiman L (2001). Random forests. Mach Learn.

[5] Cortes C, Vapnik V (1995). Support-vector networks. Mach Learn.

[6] Meyer D, Dimitriadou E, Hornik K, Weingessel A, Leisch F (2017). e1071: Misc functions of the Department of Statistics, probability theory group (formerly: E1071), TU Wien.

[7] Mandal S, Van Treuren W, White RA, Eggesbo M, Knight R, Peddada SD (2015). Analysis of composition of microbiomes: a novel method for studying microbial composition. Microb Ecol Health Dis.

[8] Lalucat J, Bennasar A, Bosch R, Garcia-Valdes E, Palleroni NJ (2006). Biology of Pseudomonas stutzeri. Microbiol Mol Biol Rev.

[9] Nemec A, De Baere T, Tjernberg I, Vaneechoutte M, van der Reijden TJ, Dijkshoorn L (2001). Acinetobacter ursingii sp. nov. and Acinetobacter schindleri sp. nov., isolated from human clinical specimens. Int J Syst Evol Microbiol.

[10] Dortet L, Legrand P, Soussy CJ, Cattoir V (2006). Bacterial identification, clinical significance, and antimicrobial susceptibilities of Acinetobacter ursingii and Acinetobacter schindleri, two frequently misidentified opportunistic pathogens. J Clin Microbiol.

[11] van Buuren S, Groothuis-Oudshoorn K. Mice : multivariate imputation by chained equations in *R*. J Stat Softw. 2011.

[12] Zou H, Hastie T. Regularization and variable selection via the elastic net. J R Stat Soc Ser B Stat Methodol. 2005.

[13] Habier D, Fernando RL, Kizilkaya K, Garrick DJ. Extension of the bayesian alphabet for genomic selection. BMC Bioinformatics. 2011. 10.1186/1471-2105-12-186 PMC3144464 21605355

[14] Höskuldsson A. PLS regression methods. J Chemom. 1988.

[15] Boulesteix A, Strimmer K. Partial least squares: a versatile tool for the analysis of high-dimensional genomic data. Brief Bioinform. 2007. 10.1093/bib/bbl016 16772269

[16] Caporaso JG, Kuczynski J, Stombaugh J, Bittinger K, Bushman FD, Costello EK (2010). QIIME allows analysis of high-throughput community sequencing data. Nat Methods.

[17] Li H, Durbin R (2009). Fast and accurate short read alignment with burrows-wheeler transform. Bioinformatics.

[18] Li H, Handsaker B, Wysoker A, Fennell T, Ruan J, Homer N (2009). The sequence alignment/map format and SAMtools. Bioinformatics.

[19] Salter SJ, Cox MJ, Turek EM, Calus ST, Cookson WO, Moffatt MF, et al. Reagent and laboratory contamination can critically impact sequence-based microbiome analyses. BMC Biol. 2014. 10.1186/s12915-014-0087-z PMC4228153 25387460

[20] Kunin V, Engelbrektson A, Ochman H, Hugenholtz P. Wrinkles in the rare biosphere: pyrosequencing errors can lead to artificial inflation of diversity estimates. Environ Microbiol. 2010. 10.1111/j.1462-2920.2009.02051.x 19725865

[21] Kim D, Hofstaedter CE, Zhao C, Mattei L, Tanes C, Clarke E, et al. Optimizing methods and dodging pitfalls in microbiome research. Microbiome. 2017. 10.1186/s40168-017-0267-5 PMC5420141 28476139

[22] Patel RK, Jain M (2012). NGS QC toolkit: a toolkit for quality control of next generation sequencing data. PLoS One.

[23] Wang Q, Garrity GM, Tiedje JM, Cole JR (2007). Naive Bayesian classifier for rapid assignment of rRNA sequences into the new bacterial taxonomy. Appl Environ Microbiol.

[24] Law Charity W, Chen Yunshun, Shi Wei, Smyth Gordon K (2014). voom: precision weights unlock linear model analysis tools for RNA-seq read counts. Genome Biology.

[25] Ritchie Matthew E., Phipson Belinda, Wu Di, Hu Yifang, Law Charity W., Shi Wei, Smyth Gordon K. (2015). limma powers differential expression analyses for RNA-sequencing and microarray studies. Nucleic Acids Research.

[26] Team RDC (2010). R: a language and environment for statistical computing.

